# Outcome of patients with soft tissue sarcomas of the extremities and trunk treated by (neo)adjuvant intensity modulated radiation therapy with curative intent

**DOI:** 10.1186/s13014-023-02238-z

**Published:** 2023-03-03

**Authors:** Hendrik Dapper, Christian Diehl, Carolin Knebel, Carolin Mogler, Kai Borm, Sophie Dobiasch, Stephanie E. Combs, Jan C. Peeken

**Affiliations:** 1 Department of Radiotherapy and Radiation Oncology, Public Hospital of Bielefeld, University Medical Center East Westphalia-Lippe, Bielefeld, Germany; 2 Department of Radiation Oncology, Klinikum Rechts der Isar, Technical University Munich, Munich, Germany; 3 Department of Orthopaedic Surgery, Klinikum Rechts der Isar, Technical University Munich, Munich, Germany; 4 Institute of Pathology, Klinikum Rechts der Isar, School of Medicine, Technical University Munich, Munich, Germany; 5 Deutsches Konsortium Für Translationale Krebsforschung (DKTK), Partner Site , Munich, Germany; 6 Institute for Radiation Medicine (IRM), Helmholtz Zentrum München, Ingolstädter Landstr. 1, Neuherberg, Germany

**Keywords:** Soft tissue sarcoma, Extremities & trunk, Intensity-modulated radiotherapy

## Abstract

**Background:**

Soft tissue sarcomas (STS) are a relatively rare group of malignant tumors. Currently, there is very little published clinical data, especially in the context of curative multimodal therapy with image-guided, conformal, intensity-modulated radiotherapy.

**Methods:**

Patients who received preoperative or postoperative intensity-modulated radiotherapy for STS of the extremities or trunk with curative intent were included in this single centre retrospective analysis. A Kaplan–Meier analysis was performed to evaluate survival endpoints. Multivariable proportional hazard models were used to investigate the association between survival endpoints and tumour-, patient-, and treatment-specific characteristics.

**Results:**

86 patients were included in the analysis. The most common histological subtypes were undifferentiated pleomorphic high-grade sarcoma (UPS) (27) and liposarcoma (22). More than two third of the patients received preoperative radiation therapy (72%). During the follow-up period, 39 patients (45%) suffered from some type of relapse, mainly remote (31%). The two-years overall survival rate was 88%. The median DFS was 48 months and the median DMFS was 51 months. Female gender (HR 0.460 (0.217; 0.973)) and histology of liposarcomas compared to UPS proved to be significantly more favorable in terms of DFS (HR 0.327 (0.126; 0.852)).

**Conclusion:**

Conformal, intensity-modulated radiotherapy is an effective treatment modality in the preoperative or postoperative management of STS. Especially for the prevention of distant metastases, the establishment of modern systemic therapies or multimodal therapy approaches is necessary.

## Introduction

Soft tissue sarcomas (STS) are a relatively rare group of malignant tumors that can occur in any part of the body [1]. Most often they are localized to the extremities. All subtypes originate from mesenchymal tissue and can be further differentiated both histologically and by molecular genetics. The most common subtypes are liposarcoma, undifferentiated pleomorphic high-grade sarcoma (UPS), leiomyosarcoma, and fibrosarcoma [1]. New pathological classifications do justice to the complexity and diversity of the subtypes of STS, which will have more and more therapeutic consequences in the future [2, 3]. Due to the great heterogeneity within the group of STS, both the extent of loco-regional spread and the probability of distant metastases differ between the subgroups. The histological subtype, tumor localization, stage, deep tissue invasion, and particularly the grade of histopathological differentiation are important tumor-specific, prognostic factors. In particular, the grading points the way to deciding on the right therapy concept [4].

STSs are usually somewhat resistant to system therapies such as chemotherapies, which is why distant metastases often occur during the course of the disease, which ultimately have a decisive influence on survival. Therefore, appropriate radical local therapy is of utmost importance for the cure of STS. Surgery in particular plays an essential role in the treatment. For highly differentiated tumors (G1), complete resection is usually sufficient.

One challenge in the curative treatment of STS is diffuse local microscopic spread and extension of the tumor to nerves and vessels. Therefore, radiotherapy plays an important role in the multimodal therapy of high-risk STS to achieve long-term local control [5]. As early as the 1980s, limb preservation after wide excision and adjuvant radiotherapy for STS of the extremities was demonstrated to have a comparable oncological outcome to amputation [7]. A large SEER database analysis was also able to show an improvement in overall survival with adjuvant radiotherapy in high-risk STS of the extremities [6]. Radiotherapy is generally equally effective both, preoperatively and postoperatively [7]. The main advantages of neoadjuvant radiotherapy are primarily in a lower radiation dose and treatment duration, smaller radiation fields and thus a lower risk of fibrosis-related late effects such as lymphoedema or osteoarthritis. The benefits of postoperative radiotherapy are knowledge of definitive margin status and histology as well as a lower rate of wound healing problems [5, 7, 8]. Intensity-modulated radiation therapy (IMRT) is a conformal radiation technique and can significantly reduce toxicity [9]. In the context of increasingly specific guidelines on target volume definition and dosage, it is important to evaluate the efficacy and prognostic factors in the treatment of STS with IMRT [10–12].

In this study, we analyzed details of radiotherapy and relevant survival endpoints, and investigated the influence of patient-, tumor-, and therapy-associated factors in the context of curatively intended (neo)adjuvant IMRT for STS treated at a nationwide sarcoma center.

## Methods

### Patients selection

This retrospective study included data from patients who received preoperative or postoperative intensity-modulated radiotherapy in curative intention for STS of the extremities or trunk which was performed at the Department of Radiation Oncology at Klinikum rechts der Isar of Technical University of Munich between 2011 and 2020. Inclusion criteria comprised STS of all histological subtypes without distant mestastases treated with curative intent with neoadjuvant, adjuvant or additive radiotherapy. Sarcomas of the bones and in other anatomical regions such as retroperitoneally located STS as well as the presence of distant metastases at initial diagnosis were excluded from the analysis. All cases were regularly discussed in an interdisciplinary tumor conference before, during, and after multimodal therapy. Histological specification, radiological diagnostics, and multimodal therapies were also carried out at a certified oncological STS center. In general, patients received at least one MRI of the primary tumor site and systemic CT or PET-CT staging at initial diagnosis.

### Data collection

Data on patient characteristics and survival endpoints or possible relapses were taken from regular, guideline-based follow-up and the original patient file. In addition, a query regarding overall survival was made to the respective registry office responsible for death registry. Tumor-specific features were determined from imaging data (staging CT, local MRI), clinical examination, and histology. All radiological and histological findings including grading were obtained at a certified institute of pathology that is part of a national sarcoma center. Histological grading was performed according to Coindre et al. using a 3-level classification scheme developed by the Fédération Nationale des Centres de Lutte Contre le Cancer (FNCLCC) [13]. Therapy-specific data, especially for radiotherapy, were obtained from the radiation planning protocols including the planning CT. Other data sources were tumor board protocols, doctor’s letters, and progress documentation.

### Statistical analysis

Kaplan–Meier analysis was performed to evaluate the endpoints of overall survival (OS), disease-free survival (DFS), distant metastasis-free survival (DMFS), and local relapse-free survival (LRFS). OS was defined as the time from initial pathologic diagnosis (date of biopsy) to death. DFS was defined as time to tumor relapse (local, regional, or distant), time to second malignancy, or time to death; DMFS was defined as time to systemic relapse or time to death; LRFS was defined as time to local or regional lymphatic relapse or time to death.

Multivariable proportional hazard models were used to examine the associations between OS and various clinical and imaging features. To avoid overfitting due to a limited number of events, we included three clinically relevant variables as covariates to define OS, LRFS, and DMFS. These were age at diagnosis, gender, and histology (UPS, Liposarcoma, myxofibrosarcoma, synovial sarcoma, others). For the definition of DFS, we observed more events and additionally included T-status, region of tumor involvement, and radiation sequence as covariates in our proportional hazards model.

All methods were performed in accordance with the relevant guidelines and regulations and informed consent was obtained from all participants and/or their legal guardians.

## Results

### Patients and tumor characteristics

86 patients met the inclusion criteria and were included in the analysis. 48 patients (56%) were men and 38 patients (44%) were women. Median age was 57 years (Q1-Q3: 41–68). The vast majority of patients had STS of the extremities (76). In only 10 cases the primary tumor was located on the trunk. The most common histological subtypes were UPS (27) and liposarcoma (22). T-Stage was mainly T2 (68; 79%) (AJCC 7^th^ edition). Only one patient with an epithelioid synovial sarcoma of the lower extremities showed regional lymph node metastases at the initial diagnosis. In the vast majority, the tumors were poorly differentiated. Histological grading based on FNCLCC was G3 in 43, G2 in 37, and G1 in six cases (Table 1).

**Table 1:** Patient-, tumor-, and treatment characteristics

Patient characteristics (n = 86)	N (%)
*Age at time of diagnosis (years)*	
Median	57
Q1–Q3	41–68
*Gender*	
Female	38 (44)
Male	48 (56)
*Follow-up (months)*	
Median	33
Q1–Q3	19–61
*Histology*	
Undifferentiated pleomorphic high-grade sarcoma	27 (32)
Liposarcoma	22 (26)
Myxofibrosarcoma	13 (15)
Synovial STS	12 (14)
Others	12 (14)
*Tumor localization*	
Extremities	76 (88)
Trunk	10 (12)
Grading (FNCLCC)	
G1	6 (7)
G2	37 (43)
G3	43 (50)
*T stage*	
T1	18 (21)
T2	68 (79)
*N stage*	
N0	85 (99)
N +	1 (1)
*Resection status*	
R0	72 (84)
R1	8 (9)
R2	1 (1)
Rx	5 (6)
*Radiation Sequence*	
Preoperative	62 (72)
Postoperative adjuvant	19 (22)
Postoperative additive	5 (6)
*RT dose*	
Preoperative median (Gy); (Q1-Q3)	50; (50–50)
Postoperative/additive median (Gy); (Q1-Q3)	60; (58–65)
*Chemotherapy*	
Yes	2 (2)
No	81 (94)
Unknown	3 (3)

*FNCLCC* Fédération Nationale des Centres de Lutte Contre le Cancer, *Gy* Gray

### Radiation therapy

Radiation planning was image-guided on the basis of a planning CT with 3 mm slice thickness after fusion with a current preoperative and postoperative contrast-enhanced MRI. Segmentation of the different target volumes was based on ICRU83.

All patients received IMRT which was performed either as helical tomotherapy or as volumetric arc therapy (VMAT) on a linear accelerator with mostly 6 MV. 62 patients (72%) were treated preoperatively, 19 patients (22%) received adjuvant, and five patients (6%) postoperative additive radiation. In the 62 patients who were treated with preoperative radiation, the median prescription dose for the CTV was 50.0 Gy (range 42.0–56.0) with 1.7–2.0 Gy daily single dose for all patients. In four cases, simultaneous integrated boost to a maximum of 56 Gy and 2 Gy single dose was performed. The CTV regularly includes the primary tumor visible on MRI (GTV) with 1–4 cm longitudinal (depending on various factors) and 1–1.5 cm radial extension including edema. 24 patients (28%) were treated postoperatively. Nine of them underwent additional therapy because microscopic or macroscopic tumor was still present after surgery. CTV1 comprised the surgical tumor bed with a longitudinal margin of 3 to 4 cm and a radial margin of 1.5 cm, including surgical scars, whereas CTV2 (boost volume) had only a longitudinal margin of 2 cm and the same radial margin, analogous to ASTRO guidelines. The median total dose in CTV2 was 60.0 Gy (50,4–66 Gy), delivered sequentially or with a simultaneous integrated boost.

Only two patients (2%) received chemotherapy, particularly in the context of treatment of rhabdomyosarcoma according to the CWS protocol.

### Survival end points

**Fig. 1:**
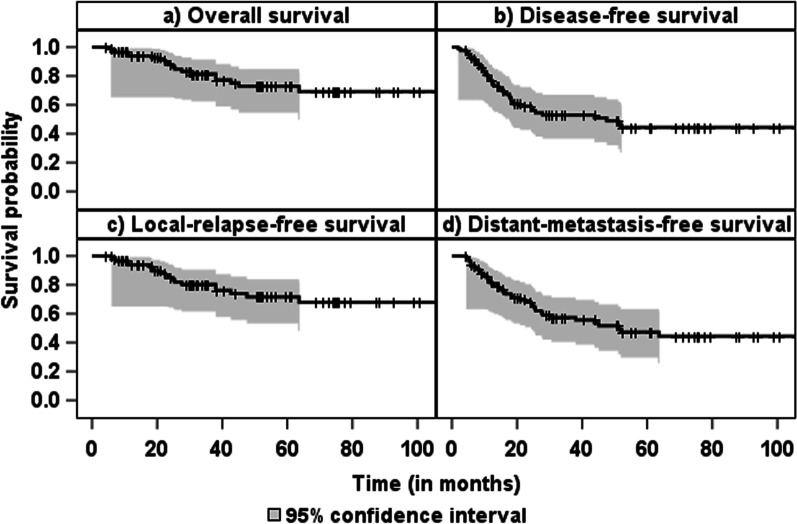
Kaplan–Maier curves of survival endpoints

The median follow-up time was 33 months (19–61). During this period, relapse occurred in 39 patients (45%). Of these patients, four had local relapse only, 27 had distant metastasis, and eight had both distant and local relapse. 18 persons (21%) died during follow-up. Of the three patients who did not undergo resection after neoadjuvant treatment, two died within a year. The third patient was lost to follow-up immediately after treatment. Two-year OS rate was 87.8% (95% confidence interval [77.8; 93.5]). The median OS and LRFS were not met. The median DFS was 48 months and the median DMFS was 51 months. Kaplan–Meier curves for OS, DFS, LRFS and DMFS can be found in Fig. 1.

### Multivariable proportional hazard model

A multivariable proportional hazard model for OS showed age (HR 1.042 (1.004; 1.080)) and male gender (HR 0.460 (0.217; 0.973)) as negative prognostic factors, with only age being statistically significant (*p* = 0.028) (Table 2). For DFS, only gender and liposarcoma compared to UPS (HR 0.327 (0.126; 0.852), *p* = 0.02) were significantly associated (Table 3). For LRFS and DMFS, only age was significant or borderline significant (Tables 4 and 5).

**Table 2:** Results of the multivariable proportional hazard model for overall survival

	Multivariable proportional hazard model	
	HR (95% CI)	*P-*value
*Gender*		
Female vs. male	0.432 (0.140; 1.332)	0.1442
Age at diagnosis	1.042 (1.004; 1.080)	0.0278
*Histology*		
Liposarcoma vs. UPS	0.366 (0.074; 1.801)	0.2163
Myxofibrosarcoma vs. UPS	1.520 (0.420; 5.498)	0.5234
Synovial sarcoma vs. UPS	0.735 (0.073; 7.360)	0.9646
Others vs. UPS	1.466 (0.377; 5.709)	0.5810

*UPS* Undifferentiated pleomorphic high-grade sarcoma

**Table 3:** Results of the multivariable proportional hazard model for disease-free survival

	Multivariable proportional hazard model	
	HR (95% CI)	*P-*value
*Gender*		
Female vs. male	0.460 (0.217; 0.973)	0.0423
Age at diagnosis	1.012 (0.987; 1.037)	0.3564
*T-Status*		
1 vs. 2	0.327 (0.088; 1.215)	0.0951
*Histology*		
Liposarcoma vs. UPS	0.327 (0.126; 0.852)	0.0222
Myxofibrosarcoma vs. UPS	0.641 (0.246; 1.670)	0.3624
Synovial sarcoma vs. UPS	0.705 (0.209; 2.374)	0.5725
Others vs. UPS	0.973 (0.346; 2.735)	0.9584
*Region*		
Trunk vs. extremities	0.657 (0.155; 2.788)	0.5688
*Radiation sequence*		
Preoperative vs. postoperative	2.126 (0.899; 5.023)	0.0857

*UPS* Undifferentiated pleomorphic high-grade sarcoma

**Table 4:** Results of the multivariable proportional hazard model for local-relapse-free survival

	Multivariable proportional hazard model	
	HR (95% CI)	*P-*value
*Gender*		
Female vs. male	0.379 (0.127; 1.127)	0.0809
Age at diagnosis	1.050 (1.010; 1.092)	0.0133
*Histology*		
Liposarcoma vs. UPS	0.481 (0.123; 1.887)	0.2942
Myxofibrosarcoma vs. UPS	1.364 (0.382; 4.880)	0.6327
Synovial sarcoma vs. UPS	0.725 (0.075; 7.047)	0.7819
Others vs. UPS	1.396 (0.359; 5.424)	0.6298

*UPS* Undifferentiated pleomorphic high-grade sarcoma

**Table 5:** Results of the multivariable proportional hazard model for distant-metastasis-free survival

	Multivariable proportional hazard model	
	HR (95% CI)	*P-*value
*Gender*		
Female vs. male	0.682 (0.336; 1.387)	0.2908
Age at diagnosis	1.023 (1.000; 1.048)	0.0542
*Histology*		
Liposarcoma vs. UPS	1.571 (0.522; 4.730)	0.4221
Myxofibrosarcoma vs. UPS	0.667 (0.186; 2.392)	0.5344
Synovial sarcoma vs. UPS	1.181 (0.323; 4.315)	0.8016
Others vs. UPS	1.675 (0.405; 6.922)	0.4759

*UPS* Undifferentiated pleomorphic high-grade sarcoma

## Discussion

We performed a comprehensive retrospective evaluation of patients with preoperative or postoperative radiotherapy for STS of the extremities and trunk at our nationwide sarcoma center. We chose these two body regions because the prognosis was comparable and radiation treatment was the same [14, 15]. For the evaluation and comparison of radiotherapy, it was important that similar target volume definitions could be used and that a certain distance to radiation-sensitive organs was ensured that did not require dose limitation. With regard to patient and tumor characteristics, our collective was largely consistent with data from epidemiological registries and studies. Men were slightly more likely to be affected than women. The average age was 60–65 years. In addition, the extremities were most commonly affected and histologically UPS or liposarcomas were the most common [16, 17]. Consistent with our findings that advanced patient age and male gender were negative prognostic factors for survival, young age and female gender were associated with better outcome [18]. The two-year overall survival as well as a median DFS were also comparable to data from studies with similar cohorts.

Unfortunately, not all desired variables such as grading could be included in the multivariable proportional hazard model because certain categories contained too few observations, making it impossible to estimate a hazard ratio. Like the resections status, tumor grading is known as one of the strongest prognostic factors [19, 20]. Adjuvant local therapy is not usually indicated for G1 tumors, except in special cases such as positive resection margins or histological subtypes such as myxoid liposarcoma that excellently respond to radiotherapy [5]. Therefore, patients with poorly differentiated tumors accounted for a large proportion of our population (93%) [15]. Unfortunately, grading and resection status could not be included in the multivariable proportional hazard model because only six patients had grade 1 disease, nine patients had positive resection margins, and certain categories contained too few observations. But of the nine patients with positive resection margins, 6 had any type of recurrence during follow up.

Radiotherapy is generally effective in improving locoregional control in STS which led to prolonged survival in large registry analyses [8, 21]. In recent years, the possibility of conformal, intensity-modulated, image-guided radiotherapy and the exploration of improved target volume definitions in STS have led to very good locoregional control rates and increasing tolerability of radiotherapy (e.g. less lymphedema or joint stiffness) [10, 14, 24]. In our analysis, twelve patients (14%) had local recurrence. 35 patients (41%) had distant metastasis during follow up. This demonstrates that in the majority of cases local tumor control can be achieved with wide resection and modern radiation technique. Analysis of a large collective of STS patients showed the particular importance of radiotherapy in relation to local relapse depending on the status of the resection margins [21–23]. Complete resection is a strong therapeutic prognostic factor for local control and overall survival in STS [24–26]. Radiotherapy was effective even in wide surgical margins [27]. However, another important fact is that R2 resection is unlikely to achieve good local control rates despite adjuvant radiotherapy [31]. Thus, radiotherapy is not a substitute for appropriate surgery. In combination with adequately applied radiotherapy, the already very good local control rates can be further increased only by excellent surgical quality. These reasons emphasize the importance of surgery in the treatment of STS and highlight the need for STS surgery to be performed in specialized and experienced centers with high surgical volumes [18].

If radiation therapy is indicated in the treatment of STS, preoperative RT is generally recommended over postoperative RT although both options are equally effective in terms of local control [10]. One of the main reasons is the need for lower radiation doses and smaller radiation fields due to good preoperative oxygenation and relatively good morphological imaging in delineating the tumor [9]. In addition, in some cases tumor downsizing can be achieved prior to surgery, which can be particularly beneficial if the tumor is located near nerves and vessels. The American Society for Radiation Oncology recommends an approach of postoperative radiation only in patients with symptoms such as extreme edema and pain or in patients in whom “the risk of wound healing complications outweighs the risk of late toxicity” which is particularly the case in very old patients with comorbidities and large surgical defects [10]. In our study, 62 patients (72%) received preoperative radiotherapy whereas only 27% received postoperative treatment. Interestingly, the hazard ratio of patients treated preoperatively compared with those treated postoperatively was 2.126 (0.899; 5.023 – not significant) which cannot be confirmed in large prospective studies that showed more of a trend toward an advantage of neoadjuvant therapy [9]. Care should be taken when retrospectively comparing neoadjuvant and adjuvant radiotherapy in STS (when there are already few cases) because patient selection for the different therapies is biased. For example, preoperative therapy may have been used primarily in patients with large tumors to shrink them and achieve complete resection. For example, in our study 85% (9) in the preoperative group had T2 disease whereas only 63% (9) had T2 disease in the postoperative group. Conversely, it could be that adjuvant therapy was administered primarily to patients with unfavorable prognostic factors such as advanced age or comorbidities predisposing to wound healing disorders.

As mentioned, large database analyses demonstrate that radiotherapy leads to significant improvement in overall survival in addition to improvement in locoregional control [8]. This may be due to the fact that radiotherapy prevents hematogenous migration of micrometastases in some cases. The overall survival of our cohort is similar or slightly higher than the results of other comparable cohorts with localized STS [18]. Other studies have also shown that approximately 40–50% of patients with STS develop distant metastases, making the prevention of hematogenous spread of soft tissue tumors particularly important [22, 24]. The histological subtype is crucial for the prognosis of STS patients. It has been demonstrated that some subtypes such as leiomyosarcomas or malignant peripheral nerve sheath tumors are particularly prone to distant metastasis [28, 29]. In our cohort, the most common subtypes were UPS (28) and liposarcomas (22). Impressively, 15 of 41 patients (37%) with distant metastases had UPS, whereas only four patients had liposarcoma. Two of these four liposarcomas were pleomorphic liposarcomas. This is not particularly surprising in that UPS sarcomas usually have a particularly pronounced degree of degeneration (G3) and therefore often cannot be clearly assigned to another histologic type. In addition, the liposarcomas are divided again into a wide variety of subgroups with very different prognosis or response to certain therapies (e.g. myxoid liposarcomas respond very well to radiotherapy) This demonstrates the importance of further molecular genetic specification of STS in order to identify the correct subtype and, if necessary, to select the adequate form of therapy.

Obviously, the frequent occurrence of distant metastases can largely only be managed with intensified or more effective systemic therapies. The dilemma is that most STS types are very chemotherapy resistant and therefore require very intensified toxic protocols. Due to the relatively low incidence and the biological heterogeneity of STS, general therapeutic concepts for the various histological subtypes are difficult to establish by large randomized trials. Only large meta-analyses have shown a significant benefit of approximately 5% in long-term 5-year survival [30–32]. In contrast, individual prospective studies showed no significant benefit [33]. Thus, a very large proportion of patients would be treated with very toxic chemotherapy with no benefit expected from the therapy. The challenge therefore is to identify suitable patients for such therapy (e.g., primarily young and fit patients with increased risk factors and suitable histology). Tools such as “SARCULATOR analysis” can help in decision making of systemic therapies in STS patients [34]. Additional biological information obtained through gene expression profiling or next generation sequencing can usefully complement these prognostic tools. In addition, because of SFS resistance to common therapies advanced multimodal therapies should be explored. A randomized phase III study showed a benefit in OS for G2/3 (FNCLCC) localized STS (≥ 5 cm) that received chemotherapy plus hyperthermia. Compared with patients who received neoadjuvant chemotherapy alone, these patients had significantly better survival rates (5-year survival of 63% vs. 51%) [8]. Most patients also received radiotherapy. The complexity of accounting for histologic subtypes, the relatively low incidence, and the treatment of expected severe toxicities highlight the urgent need for treatment in advanced STS centers, which are not available nationwide.

Unfortunately, neoadjuvant chemotherapy protocols tailored to histology appear to be more detrimental compared with standard anthracycline plus ifosfamide therapy [42]. Thus, in addition to multimodal therapies with hyperthermia, the future of systemic therapies may lie more in potentially targeted therapies. The increasing molecular genetic differentiation of STS allows the use of specific or targeted therapies in more and more cases [35]. For example, the detection of special mutations of receptor tyrosine kinases allows the promising use of tyrosine kinase inhibitors such as Pazopanib in certain patients [36]. This therapy seems to make sense especially for elderly patients due to better tolerability. A non-inferiority could be shown at least in metastatic disease in elderly patients (> 60 years) in a phase II study [37].

The extent to which combined targeted drug therapy affects the effectiveness and severity of side effects of additional radiotherapy in STS patients is not yet known and should be investigated in the future.

## Conclusion

Conformal, intensity-modulated radiotherapy with daily imaging is an effective treatment modality in the preoperative or postoperative treatment of soft tissue STSs. Histology constitutes an important predictor for disease-free survival. Especially for the prevention of distant metastases, the establishment of modern system therapies or multimodal therapy approaches is necessary.

### Limitations

Due to the low incidence of soft tissue STS, the cohort was quite small and there were many censorships during the follow-up period. Because this was a retrospective study, it was necessary to adjust for potential confounders, which was done using a proportional hazard model. However, the small sample size and infrequent occurrence forced us to limit the number of confounding variables included to a reasonable level.

## Data Availability

All data generated or analyzed during this study are included in this published article.

## Abbreviations

CTV: Clinical target volume
CTCAE: Common terminology criteria for adverse events
DFS: Disease-free survival
DMFS: Distant metastasis free survival
GTV: Gross tumor volume
ICRU: International commission on radiation units
IGRT: Image-guided radiotherapy
IMRT: Intensity-modulated radiotherapy
LRFS: Local-relapse-free survival
MRI: Magnetic resonance imaging
OS: Overall survival
PTV: Planning target volume
RFS: Relapse-free survival
RTOG: Radiation therapy oncology group
SIB: Simultaneous integrated boost
STS: Soft tissue sarcoma
